# The Essentials of Bandaging

**Published:** 1883-07

**Authors:** 


*The Essentials of Bandaging.* By BERKELEY HILL, M.B. Lond., F.R.C.S. Fifth Edition, pp. 341. London: Smith, Elder & Co., 1883.

The new edition of this favourite little work has been improved by special additions on Ophthalmoscopy by Mr. John Tweedy, and on Laryngoscopy by Dr. Vivian Poore; while Mr. Bailey has revised the instructions for administering Anæsthetics. There is an excellent description of Lister's method of dressing wounds; and in other respects the work is fairly well up to date. But it is not yet perfect. The author surely does not properly estimate the relative value of things, when among all the improvements in surgical apparatus and appliances brought in since the fourth edition was published, he should content himself with introducing "accounts of Thomas's splints, of Croft's plaster of Paris splints, and of Carr's splints;" and specially notify these additions in the preface.

The least satisfactory part of the work is that on Dislocations. Take "Backward Dislocation of the Elbow Joint" for instance. In reducing this dislocation "the patient sits on a chair on which the surgeon rests his foot, pressing his knee against the forearm at the elbow for a fulcrum; then, grasping the wrist with one hand, and steadying the arm with the other, he flexes the elbow to dislodge the coronoid process from the fossa at the back of the humerus; when this is done, the articulating surfaces slip into place." Overlooking the bad grammar and the involved description, we doubt if this is trustworthy as a statement of fact. Not a word is said of what many surgeons consider the best of all methods for reducing this dislocation—that by over-extension. In the reduction of hip dislocations the student might conclude from the description in the text that the method by pulleys still held the prominent position it did twenty years ago. Reduction by manipulation may be successful only "when the patient is not very muscular, and the dislocation recent," he says.

The best part of the work is the long chapter on Surface-guides and Landmarks.

The lists of instruments given as necessary for the various operations are at least extensive. We doubt if many surgeons carry a bull's-eye lantern with them to perform an ovariotomy, or an Otis's coil refrigerator for a circumcision. And yet, in the operation for transfusion of blood, Aveling's apparatus is not even mentioned.

